# When Ultra-Processed Foods are Eaten: The Missing Dimension in Exposure Measurement for Obesity Research

**DOI:** 10.1007/s13679-026-00765-w

**Published:** 2026-09-18

**Authors:** Jimmy Chun Yu Louie

**Affiliations:** Department of Biomedical, Health and Exercise Sciences, School of Health Sciences, Swinburne University of Technology, Hawthorn, VIC Australia

**Keywords:** Ultra-processed food, Obesity, Chrononutrition, Dietary exposure assessment, Measurement error, Causal inference

## Abstract

**Purpose of Review:**

Almost every study linking ultra-processed foods (UPFs) to obesity measures exposure as the proportion of energy or weight derived from Nova group 4 foods, averaged across days. This scalar is commonly treated as though it were the exposure itself, yet it embodies an untested assumption: that the timing and context of intake carry no information beyond total amount. This review examines what is lost when occasion-level intake is collapsed to a daily average and how that loss may affect epidemiological inference.

**Recent Findings:**

Controlled circadian-misalignment protocols and time-restricted eating trials show that the metabolic response to a fixed quantity of food varies with its timing. Postprandial studies further show that meal sequence, co-ingestion and eating-occasion structure modify glycaemic and appetite responses at equal daily totals. These findings expose a mismatch between the timescale at which relevant processes operate and the daily scale at which UPF exposure is conventionally measured. Measurement-error and causal-inference frameworks show how such temporal collapse could attenuate or otherwise distort effect estimates, reduce transportability and obscure the intervention to which an association refers.

**Summary:**

The daily group 4 fraction is a modelling choice, not a complete representation of the diet. It discards temporal and contextual structure that may explain heterogeneity across UPF-obesity cohorts and determine the causal contrast represented by their estimates. This review proposes a time-resolved, context-aware construct and specifies the studies needed to test whether it adds information beyond the conventional scalar. The controlled timing literature manipulated total energy, macronutrients or glucose loads rather than Nova group 4 foods, so its extension to UPFs remains a testable inference rather than an established finding. The immediate priority is therefore not to replace the scalar, but to analyse cohorts whose dietary records retain item-level time stamps and determine whether the discarded structure predicts outcomes beyond total amount.

## Introduction

Ultra-processed foods (UPFs) have moved rapidly from taxonomic concept to policy target. Almost two decades after the Nova classification introduced a scheme grouping foods by the nature and extent of industrial processing [1, 2], its fourth group has become the organising exposure for a literature linking diet to obesity, type 2 diabetes, cognitive and mental disorders, cardiovascular disease and mortality [3–8]. A controlled inpatient feeding trial gave the association experimental weight, showing that an ad libitum ultra-processed diet drove higher energy intake and weight gain against a minimally processed comparator matched for presented macronutrients [9]. Dietary guidelines now reference UPFs [10], fiscal and regulatory instruments are under active discussion [11, 12], and the World Health Organization has initiated formal guideline development on UPF [13]. Guideline development requires an exposure definition precise enough to support actionable thresholds. The adequacy of the current metric is therefore a policy question, not merely an academic one.

Underlying this rapid translation is a striking uniformity of measurement. Across cohorts, trials and policy analyses, foods are coded into Nova groups: the energy or weight from group 4 items is summed, expressed as a daily proportion, and averaged across assessment days [1, 14]. The resulting scalar enters models as the exposure of interest. This procedure is efficient, but it embeds a consequential assumption: that any information contained in the timing and context of group 4 intake is either negligible or fully captured by daily amount. Whether that assumption is tenable for the mechanistic, epidemiological and policy questions now being asked is the focus of this review.

Three developments now make that assumption testable. First, controlled circadian studies show that identical nutrient loads can produce different responses depending on their biological timing [15, 16]. Second, continuous glucose monitoring and postprandial metabolomics can resolve responses at the level of individual eating occasions [17, 18]. Third, measurement-error and causal-inference frameworks provide the means to determine whether collapsing occasion-level structure into a daily scalar alters effect estimates or obscures the intervention they represent [19–21]. Together, these developments permit the standard metric to be evaluated rather than simply accepted.

*Scope and demarcation.* Most criticism of Nova group 4 exposure has concerned category composition: heterogeneous products are assigned to the same group [22, 23], additives are recorded by neither identity nor dose [24, 25], and prevalence estimates vary with coding conventions [26, 27]. A companion review develops that compositional critique and argues for attribute-aware refinement [28].

The present review addresses a different problem: the aggregation applied after foods have been classified. Even if every food were assigned to group 4 without error and the category were compositionally homogeneous, intake would still be summed within days and averaged across days before analysis. The compositional question is which foods count. The present question is which properties of their consumption survive aggregation. The first concerns category membership, whereas the second concerns the arithmetic applied to that category. Correcting either problem would not correct the other. Accordingly, this review holds classification constant and asks whether the standard scalar preserves the temporal and contextual dimensions relevant to obesity. It does not reassess the biological pathways linking UPFs to appetite and body weight [28, 29], except to consider the timescale at which they are proposed to operate. Nor does it reappraise whether UPFs have a causal role in obesity, which has been reviewed elsewhere [30]. The argument is narrower:* does the dominant metric adequately represent the exposure on which that causal literature depends?*

*What is new here.* Studies of late eating and irregular meal patterns already treat timing as an exposure associated with adiposity [31, 32]. The argument advanced here is different. Timing and eating context may be properties of UPF exposure itself, rather than merely covariates accompanying it. If so, the conventional daily fraction is not simply a noisy measure of a complete exposure; it is an incomplete specification that cannot represent how group 4 intake is distributed across biologically distinct occasions.

This distinction determines the analytical response. If timing is an independent modifier, the conventional UPF variable can be retained and timing added as a covariate or interaction term. If timing is part of the exposure, adjustment leaves the original variable incompletely specified and the exposure must instead be reconstructed. The central claim of this review is therefore metrological rather than aetiological: the field has never directly tested whether the daily group 4 fraction adequately represents the exposure it purports to measure. The remainder of this review is organised around that question. It first examines whether the standard metric omits biologically meaningful structure and then asks what evidence would be required to show that retaining that structure improves inference.

The review first describes the evidence base and the scalar instrument on which it rests. It then asks whether that instrument captures variation at the timescale at which relevant processes operate. It next identifies the temporal and contextual structure removed by aggregation and traces the consequences for measurement and causal interpretation. The final sections specify a time-resolved construct, the analyses required to validate it, and the implications for feasibility, equity and obesity practice. Box 1 defines terms used across chrononutrition, epidemiology and causal inference.

Box 1. Glossary

**Table:** 

**Chrononutrition:** the study of how the timing of food intake interacts with the circadian system to influence metabolism, energy balance and disease risk
**Circadian misalignment:** a mismatch between the timing of behaviour, including eating and sleeping, and the phase of central and peripheral circadian clocks, as occurs in shift work
**Eating-occasion architecture:** the number, spacing and type (meal, snack or replacement) of discrete eating events across a day
**Estimand:** the quantity a study aims to estimate, defined by the causal contrast and target population and specified before analysis
**Target-trial emulation:** an analytical strategy in which an observational analysis is designed to mimic the randomised trial that would have answered the causal question, by specifying eligibility, treatment strategies, assignment, follow-up and outcome in advance, thereby making the estimand and its assumptions explicit
**Exposure misclassification:** assignment of an incorrect exposure value. Non-differential misclassification, unrelated to the outcome, typically attenuates associations towards the null; differential misclassification can bias estimates in either direction
**Nova group 4:** the ultra-processed category of the Nova classification, comprising industrial formulations with ingredients and additives not typically used in home kitchens
**Time-restricted eating:** confinement of daily energy intake to a defined window, studied as a circadian-aligned strategy

## Methods

This is a narrative review, and the synthesis is interpretive rather than systematic. Literature was identified through PubMed, Scopus and Web of Science searches combining terms for ultra-processed food and the Nova classification with terms for meal timing, chrononutrition, circadian rhythm, time-restricted eating, eating occasions, dietary assessment, measurement error and causal inference, supplemented by citation chaining from key papers and by hand-searching the reference lists of recent reviews. Searches covered publications to June 2026, with priority given to work published since 2020 and to primary studies over secondary syntheses when both were available. Older publications were retained where they established a method, a definition or a finding that later work builds upon, and these are identified as such. Because the aim is to appraise a measurement instrument rather than estimate an effect, no formal quality assessment or quantitative synthesis was undertaken, and no effect estimates are pooled. Selection of studies for discussion reflects my judgement of their bearing on the argument, which is the principal limitation of this format.

## The Evidence Base

### Epidemiological and Experimental Evidence

The association between UPF intake and obesity is one of the most consistently replicated findings in nutritional epidemiology. An umbrella review of meta-analyses reported convergent associations across dozens of outcomes, with obesity and cardiometabolic disease among the most consistent [5]. Prospective cohorts on several continents report dose–response relationships with weight gain and adiposity [3, 33, 34], and with incident type 2 diabetes and cardiovascular disease [35, 36]. The reach of the exposure continues to widen, with recent syntheses extending it to cognitive outcomes [8], and the proponents of the framework have restated the central thesis and its supporting evidence in detail [37].

Observational findings cannot alone separate processing from the nutrient profile and dietary patterns accompanying it. The inpatient trial of Hall et al. [9] is therefore pivotal: under residential conditions with diets matched for presented energy and macronutrients, participants consumed roughly 500 kcal per day more on the ultra-processed arm and gained weight. These findings suggest that characteristics beyond average nutrient composition promote overconsumption. Caution is warranted in extrapolating from a short trial to long-term weight trajectories [38, 39], and a recent appraisal concluded that although the observational and clinical evidence is consistent, mechanistic understanding remains inconclusive [30].

## The Instrument and the Information it Discards

### Construction of the Standard Scalar

UPF exposure assessment begins with 24-h recalls, food records or food frequency questionnaires [40, 41]. Reported foods are assigned to Nova groups, after which the energy or weight from group 4 items is summed, divided by total intake, and averaged across days [14]. Classification introduces uncertainty through database granularity, mixed-dish treatment and coder judgement [23, 26, 42], but that uncertainty is analytically distinct from temporal aggregation. Any time-resolved construct should therefore be derived from the same coded records as the conventional scalar. Holding classification constant ensures that any incremental value is attributable to retained temporal information rather than to differences in recoding.

### Mismatch Between Measurement Scale and Mechanistic Timescale

The adequacy of any summary measure depends on whether it preserves variation at the scale relevant to the outcome. The central question is therefore not whether aggregation occurs, but whether a daily average retains the timescale at which the proposed effects of UPFs operate.

Several proposed mechanisms operate within individual eating occasions Table 1. Energy density and food form influence the amount consumed before satiation develops [43, 44], while texture and oral processing affect eating rate occasion by occasion [28, 45, 46]. Glycaemic and insulinaemic responses depend both on the composition of an occasion and on the circadian phase in which it occurs [47, 48]. Other proposed pathways, including microbial, intestinal-barrier, hypothalamic and adipose signalling, are more plausibly shaped by repeated exposure patterns over longer periods [28, 29].

**Table 1:** Proposed mechanisms linking ultra-processed foods to obesity, audited by the timescale at which each operates

Mechanism	Timescale of action	Registered by a daily group 4 average?	Key references	Feasible digital measurement
Energy density and food form	Single eating occasion	Partly; amount only, not distribution	[43, 44]	Image-assisted recall; wearable ingestion sensors
Eating rate and oral processing	Single eating occasion	No	[45, 46]	Wearable chew and bite sensors; video-coded meal tests
Glycaemic and insulinaemic load	Occasion, conditional on circadian phase	No	[47, 48]	Continuous glucose monitoring
Meal sequence and co-ingestion	Within-meal	No	[59, 60]	Time-stamped recall with item order
Food matrix disruption	Food and meal	Indirectly, through Nova class	[61]	Not directly measurable; inferred from product coding
Gut microbial and barrier disruption, hypothalamic inflammation, adipose signalling	Chronic patterning	Partly; poorly by a 3–4 day average	[28, 29]	Stool sampling and metabolomics; not occasion-resolved

Mechanistic detail is reviewed elsewhere [28, 29]; this table asks only whether a daily average exposure can register each mechanism.

The conventional daily fraction aligns imperfectly with both scales. It does not retain the timing, composition or sequence of individual occasions, yet an average across a small number of assessment days also represents chronic patterning only approximately. The central measurement problem is therefore one of scale mismatch. The exposure is summarised at the level of daily averages, whereas several of its proposed effects operate at the level of individual occasions and others at the level of longer-term patterning. The question is whether that mismatch discards information relevant to the outcome.

One qualification applies to this audit. The assigned timescales are inferred largely from studies that did not manipulate Nova group 4 foods. The table therefore identifies where each mechanism would be expected to operate if relevant to UPFs, not where it has been demonstrated to operate specifically for them.

### Structure Removed By Aggregation

**Fig. 1:**
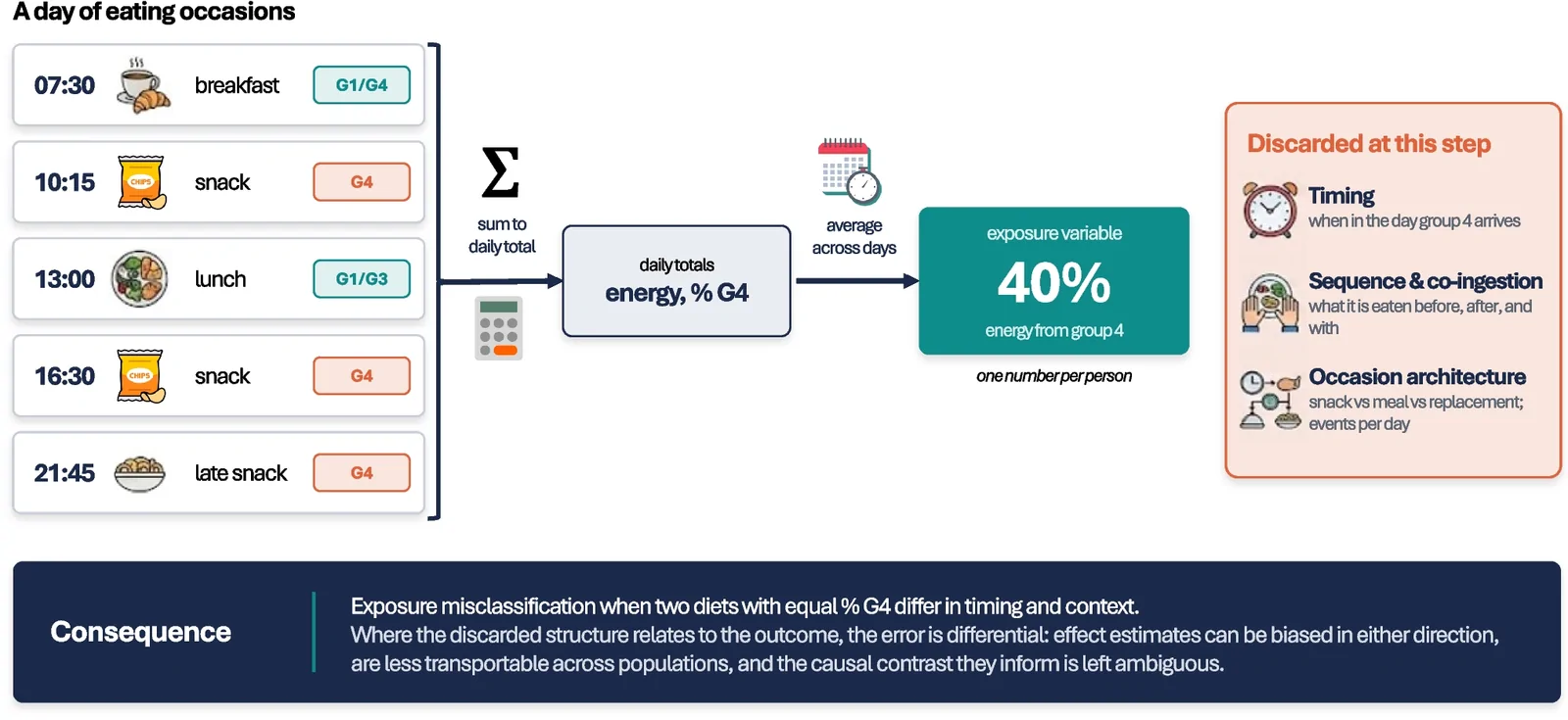
From time-resolved intake to a collapsed scalar, and what is discarded. A day of eating occasions, each carrying clock time, Nova classification, energy and meal context, is summed to a daily total and averaged across days into a single exposure value. The schematic marks the three dimensions removed at this step, namely timing, sequence and co-ingestion, and occasion architecture, and links their removal to exposure misclassification and, where the discarded structure relates to the outcome, to effect estimates that can be biased in either direction, are less transportable across populations, and inform an ambiguous causal contrast. G1, unprocessed or minimally processed; G3, processed; G4, ultra-processed

Aggregation is not inherently problematic. Daily energy intake is itself an aggregate, and that summary is meaningful because energy balance integrates over time. The relevant test is whether the outcome depends only on the total or also on its distribution. For UPF exposure, the conventional collapse removes three classes of structure Fig. 1.

First, it removes timing. Although recalls commonly retain the clock time of each eating occasion, this distribution is integrated out when group 4 intake is summed within days and averages across days. Second, it removes sequence and co-ingestion. Group 4 items are consumed alone or alongside fibre, protein and fat, and meal order can alter glycaemic and hormonal responses. Third, it removes eating-occasion architecture, including whether intake occurs within structured meals, as isolated snacks, or across repeated episodes of grazing.

These data are not necessarily absent from the instrument. In time-stamped recalls and food records, they are collected and subsequently removed during exposure derivation. Temporal collapse is therefore a potentially reversible analytical decision, not an unavoidable limitation of dietary assessment. Whether the discarded structure matters depends on whether equal daily amounts can represent different physiological exposures. Timing provides the clearest test of that proposition and is considered first.

## The Temporal Dimension

Timing provides the clearest test of the argument because its effects have been examined experimentally. The relevant question is not whether late eating is independently associated with obesity, but whether equal quantities of food can produce different responses when consumed at different biological times. If they can, a daily exposure metric that is invariant to timing cannot fully distinguish the exposures being compared.

### Circadian Control of Metabolism

**Fig. 2:**
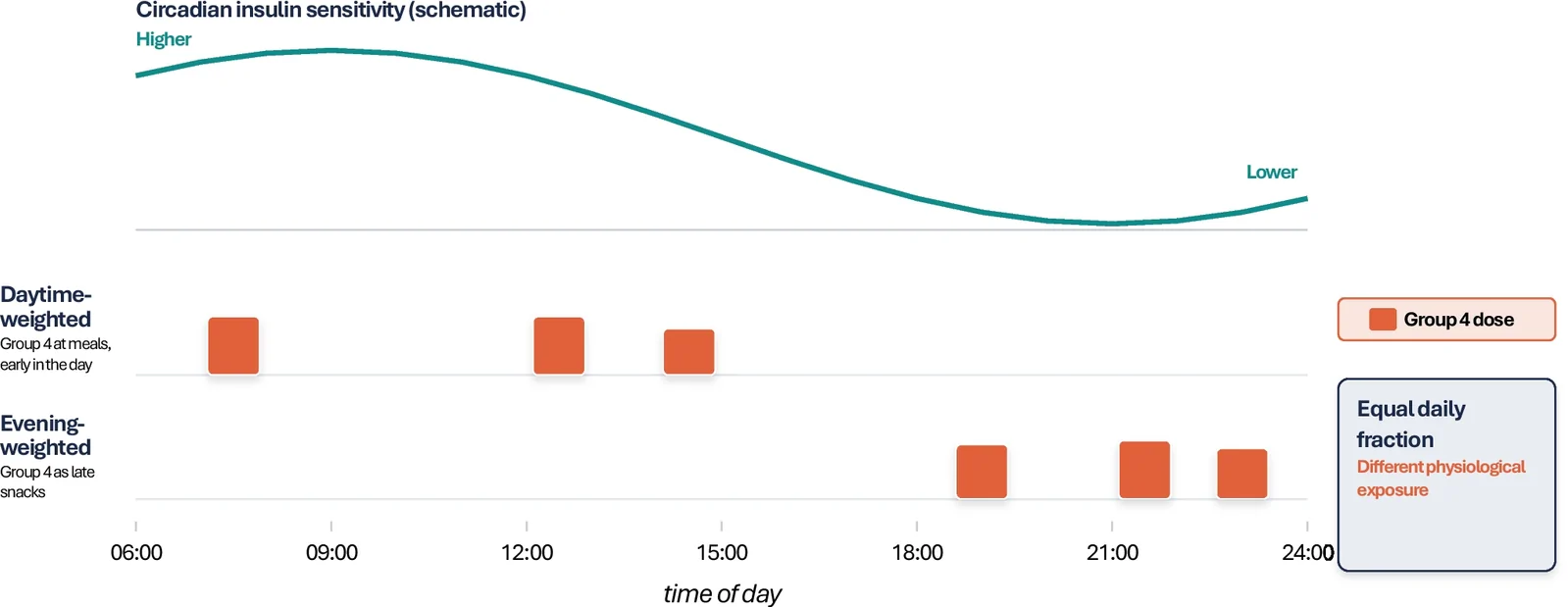
The same daily dose meets a different metabolic state depending on timing. A fixed daily quantity of group 4 energy distributed across the waking day in a daytime-weighted and an evening-weighted pattern. Set against a schematic circadian curve of insulin sensitivity, higher earlier in the biological day and lower in the late evening, the two patterns encounter the metabolic system in different phases, so equal scalar exposures correspond to different physiological exposures. The curve represents the direction of circadian variation rather than measured values and is not drawn to scale

Glucose tolerance, insulin sensitivity, insulin secretion and the thermic effect of food vary systematically across the circadian cycle [48, 49]. As a result, identical nutrient loads can produce different physiological responses depending on when they are consumed relative to biological time. Figure 2 illustrates how a fixed daily quantity of group 4 energy meets this varying metabolic state differently depending on its distribution across the waking day.

### Experimental Evidence

The strongest evidence comes from controlled misalignment protocols, in which participants eat and sleep at adverse internal clock times. Misalignment raises postprandial glucose and reduces insulin sensitivity with intake held constant, showing that timing modulates response independently of amount [15, 16]. Feeding and field studies converge, with effects extending beyond glycaemia to body weight itself. In a weight-loss cohort, late eaters lost significantly less weight than early eaters despite equivalent energy intake, appetite and sleep duration [50]. In an isocaloric crossover, loading energy at breakfast rather than dinner produced greater weight and waist reduction alongside better glycaemic and lipid outcomes [51]. Controlled chamber work suggests that appetite regulation contributes importantly to these effects [52]. Late chronotype and late meal timing have in parallel been associated with higher adiposity in observational work [31]. These studies indicate that the physiological and clinical consequences of intake depend partly on when it occurs, independently of amount. Meal timing and frequency have accordingly been recognised as cardiometabolic considerations in scientific statements [32]. However, the studies manipulated total energy, macronutrients or glucose loads rather than Nova group 4 foods. They therefore establish a timing effect for food in general, not for UPFs specifically. Whether the same differential applies to Nova group 4 foods, and whether it is larger or smaller in magnitude, remains unknown and is considered in Sect. "Limits of Inference From General Timing Studies to UPFs".

### Time-Restricted Eating

Time-restricted eating, which confines intake to a defined window without necessarily changing amount, offers a complementary line Table 2. Early windows have improved insulin sensitivity, blood pressure and oxidative stress markers, in some studies without weight loss [53, 54]. Larger trials and syntheses report more modest or weight-loss-mediated effects, and debate continues over the size and durability of timing-specific benefit [55–57]. Resolving that debate is not required here. Across this literature, the metabolic response to a fixed quantity of food depends partly on when it is consumed.

**Table 2:** Representative experimental evidence that the timing of intake modulates metabolic response

Study type	Manipulation	Principal finding	Reference
Circadian misalignment, laboratory	Eating and sleeping at adverse internal clock times, intake controlled	Higher postprandial glucose, lower insulin sensitivity	[15, 16]
Weight-loss cohort	Earlier versus later main meal	Earlier eaters lost more weight at matched intake	[50]
Isocaloric crossover	Calories loaded at breakfast *vs*. dinner	Greater weight loss and better glycaemia with breakfast loading	[51]
Early time-restricted feeding	Eating window confined earlier	Improved insulin sensitivity and blood pressure without weight loss	[53]
Time-restricted eating, metabolic syndrome	10-h eating window	Reduced weight, blood pressure, atherogenic lipids	[54]

No listed study examined Nova group 4 foods specifically; all manipulated the timing of energy or macronutrients, so extension to ultra-processed foods is an inference (Sect. "Limits of Inference From General Timing Studies to UPFs").

### Limits of Inference From General Timing Studies to UPFs

Two inferential steps require separation. First, clock time is only a proxy for internal circadian phase, which varies with chronotype, sleep timing and work schedule [31, 58]. A meal eaten at the same clock hour may therefore occur at different biological times in different individuals. Timing features should, where possible, be interpreted relative to an estimate of circadian phase.

Second, the controlled evidence reviewed above concerns the timing of total energy, macronutrients or glucose loads, rather than group 4 foods. It establishes that metabolic response depends partly on when food is consumed, but it does not establish the magnitude or direction of that differential specifically for UPFs. Two competing hypotheses are plausible. Rapid digestibility, high energy density and low satiety per unit energy could amplify the disadvantage of consumption during a circadian phase characterised by lower insulin sensitivity. Conversely, hyper-palatability could make intake comparatively insensitive to internal metabolic state, attenuating the relative importance of timing. Neither prediction has been tested directly.

The temporal argument should therefore remain conditional. A diet in which group 4 intake is concentrated late represents a different temporal exposure from one in which the same amount is concentrated early, yet the conventional scalar assigns them the same value. Whether that distinction improves prediction or causal explanation is the empirical question addressed by the construct proposed below.

### Re-referencing Timing for Shift Workers and Non-Standard Schedules

The distinction between clock time and biological time becomes most consequential in shift-working populations. Shift workers present a particular challenge for this framework. They are among the populations in whom temporal disruption is likely to be most important, yet they are also the group for whom the construct, as currently specified, performs least well. All features defined on clock time implicitly assume that clock time approximates biological time. For workers adapted to a nocturnal schedule, that assumption need not hold. A meal consumed at 02:00 may occur during the biological afternoon of an adapted night worker but during the biological night of a day-active individual. Similarly, an evening-share metric based on an 18:00 threshold has little meaning for someone whose habitual wake time is 16:00. Applying the construct without modification would therefore introduce systematic misclassification in precisely the population where accurate temporal classification is most important.

The solution is to re-reference the construct from external to internal time. Rather than defining features by clock hour, they should be calculated relative to biological phase, most simply as hours elapsed since habitual wake. This approach requires only sleep-timing data and is feasible in most cohorts that collect sleep questionnaires. Under this framework, the evening-share analogue becomes the proportion of Nova group 4 energy consumed during the final third of the habitual waking period, while the centroid becomes a phase-relative rather than a clock-relative measure. In predominantly day-active populations, these features reduce to their clock-time equivalents, allowing a common conceptual framework across populations.

Where greater precision is required, biological phase should be estimated objectively. Dim-light melatonin onset remains the reference standard for circadian phase assessment and is feasible in small mechanistic studies, although its cost and participant burden limit its use in large cohorts. Wrist actigraphy offers a practical intermediate option by providing rest-activity phase markers suitable for moderate sample sizes. For large epidemiological studies, mid-sleep timing on free days provides an inexpensive questionnaire-based proxy, albeit with substantial individual-level error. Adaptation status also warrants consideration. A worker following a permanent night schedule may exhibit substantial phase shifting, whereas a worker on a rapidly rotating schedule is often only minimally adapted. Despite sharing the same occupational label, these groups may require different analytical treatment.

Two analytical implications follow. First, studies including shift workers should report the phase proxy used and compare clock-relative with phase-relative specifications. Divergence between the two estimates would indicate the extent to which external clock time misrepresents the relevant temporal exposure. Second, shift workers should not be excluded merely to preserve a clock-time definition. Doing so would remove the population in whom temporal misclassification may be greatest and would narrow the evidence base along socially patterned occupational lines.

## The Contextual Dimension

Timing alone is sufficient to motivate the measurement critique developed here. Contextual factors represent a second category of discarded information whose evidential basis is less developed but mechanistically plausible.

Within eating occasions, the response to a group 4 item depends partly on the matrix in which it is consumed. Controlled crossover studies show that food order can alter postprandial glucose and insulin responses [59, 60], while food structure and matrix influence rates of digestion and nutrient absorption [43, 61]. A sweetened beverage consumed alone and the same beverage consumed with a mixed meal containing fibre, protein and fat therefore make identical contributions to the daily group 4 fraction but need not constitute identical physiological exposures.

Eating-occasion architecture may create a related distinction. Consuming a given quantity of group 4 food across repeated snacks generates more discrete postprandial episodes than consuming it within fewer composite meals and may produce different patterns of appetite and subsequent compensation [32, 62]. The scalar retains the total contribution but not whether it occurred as a meal component, a meal replacement or a series of isolated snacks.

Substitution is conceptually related but analytically distinct. A group 4 item added to an unchanged diet and one displacing fruit or another food may have different net effects, even if they produce the same daily fraction. Because substitution requires an explicit replacement contrast and has been treated elsewhere [25], it is not incorporated into the construct developed here.

The temporal and contextual dimensions share the feature relevant to this review: each can vary between individuals with the same daily group 4 fraction, and each is removed when occasion-level records are collapsed. The evidence is strongest for timing and more provisional for sequence, co-ingestion and occasion architecture. The proposed construct preserves both so that their incremental value can be tested separately rather than presumed collectively.

## From Discarded Structure to Distorted Inference

### Exposure Misclassification

The preceding sections establish that equal daily group 4 fractions can conceal meaningfully different exposures. The statistical question is what follows when those differences are ignored. The omission is measurement error with structure Fig. 1. Two individuals with the same daily group 4 share but different temporal patterns have different true exposures, and the metric assigns them the same value [63, 64].

A worked comparison makes the mechanism concrete. Consider two adults, each deriving 40% of daily energy from group 4 foods. The first takes that contribution as a fortified breakfast cereal at 07:30 and a sandwich at 13:00, both within composite meals containing fibre and protein. The second takes an identical contribution as a confectionery item at 16:00 and a packaged snack at 22:30, each eaten alone. Every dimension the metric records is identical. Every dimension it discards differs: circadian placement, co-ingestion, occasion type and the number of separate postprandial events. The two are assigned the same exposure value and treated as exchangeable in every analysis that follows, although the physiology they experience is not the same.

Under classical measurement-error assumptions, non-differential error in a continuous exposure generally attenuates associations towards the *null* [65, 66]. That remains the appropriate baseline expectation for the conventional UPF scalar. A stronger claim, that temporal collapse produces systematic bias whose direction may differ across populations, requires evidence that the omitted temporal component is associated both with the measured amount and with the outcome.

Those conditions are plausible but have not been demonstrated for UPF exposure. Timing is associated with metabolic outcomes, and group 4 intake may cluster at particular times, but no quantitative bias analysis has established the magnitude or direction of bias produced by collapsing the two. Non-differential attenuation should therefore remain the working assumption unless analysis shows otherwise. Sect. "Specification of a Quantitative Bias Analysis" specifies the analysis required to determine when temporal collapse behaves as simple information loss and when it produces systematic omitted-variable bias.

### Heterogeneity, Confounding and The Causal Question

This measurement structure has three important consequences. Reported effect sizes are conditional on a population's joint distribution of amount and timing, so they need not transport across populations whose eating schedules differ [67]. A cohort in which group 4 intake clusters at breakfast and one in which it clusters late could report different effect sizes for the same daily fraction, a divergence driven by temporal exposure rather than by processing, which adds to the heterogeneity visible across UPF-obesity cohorts. Residual confounding by temporal pattern is plausible wherever late or fragmented eating travels with short sleep or shift work, and the scalar cannot adjust for what it does not record [68]. And mechanistic interpretation suffers, since an association attributed to processing may partly reflect the timing and context in which these foods are habitually eaten.

Improved measurement also clarifies which intervention an association can inform [20]. Reducing the amount of group 4 intake, shifting it to an earlier biological phase and changing the context in which it is consumed are distinct interventions. Each requires a separate estimand that specifies which dimension changes and which remain fixed. The conventional scalar cannot distinguish these contrasts, so its coefficient has ambiguous interventional meaning. A time-resolved construct would permit amount, timing and context contrasts to be defined separately and, where assumptions of intervention consistency, exchangeability and positivity are credible, estimated within an explicitly specified target-trial framework [20, 69].

### Specification of a Quantitative Bias Analysis

Whether temporal collapse causes only attenuation or generates systematic omitted-variable bias cannot be determined conceptually. It requires quantitative bias analysis. The following simulation is specified so that the hypothesis can be implemented and tested independently. Let *A* denote daily group 4 energy fraction, *T* the temporal distribution of that intake, summarised by a feature such as evening share, and *Y* an outcome such as change in body mass index during follow-up.

The first step is to specify the joint distribution of *A* and *T* in a source population. Wherever possible, this should be estimated from cohorts that retain item-level timestamps rather than assumed, because the association between amount and timing is likely to be a major determinant of any resulting bias. The second step is to define the outcome model:

$$Y=f\left(A, T\right)+ \varepsilon$$

assigning *T* an effect size informed by the chrononutrition literature and varying that effect across a plausible range, including zero as the *null* case. The simulation tests a single causal structure: timing affects the outcome, and timing is associated with measured UPF amount. If either condition fails, systematic omitted-variable bias cannot occur.

The third step is to generate the observed exposure by applying the conventional collapse, retaining *A* while discarding the temporal component *T*. The fourth step is to fit the conventional model, regressing *Y* on *A*, and record the resulting estimate. The fifth step is to compare that estimate with the corresponding parameter, or marginal effect, in the data-generating model. The difference provides an estimate of the bias attributable to temporal collapse under the specified parameter values. Under a linear additive model, this comparison reduces to the coefficient on *A*. When interactions or nonlinearities are present, the estimand should be defined accordingly.

The analysis becomes informative when repeated across a sensitivity grid. This grid should vary the correlation between *A* and* T*, because omitted-variable bias requires both an effect of *T* on *Y* and an association between *T* and *A*. As this association approaches zero, omission of *T* increasingly resembles the loss of independent information rather than a source of systematic bias. The grid should also vary the magnitude of the timing effect, the reliability of *T* as discussed in Sect. "Measurement error in temporal and contextual features", and the functional form of *f*. Reliability matters because measurement error in *T* attenuates its observed association with the outcome and may therefore alter the apparent consequences of temporal collapse. Analyses that examine reliability should distinguish between the latent timing construct and its measured representation. Particular attention should be given to models containing *A* × *T* interactions, as departures from additivity are likely to amplify the consequences of collapse. Reporting the resulting bias surface, rather than a single estimate, would identify the conditions under which temporal collapse attenuates, inflates, or leaves the estimated effect approximately unbiased.

The prespecified hypothesis is that temporal collapse will produce little systematic bias when *A* and *T* are weakly associated, although loss of independent temporal information may still reduce predictive performance. Bias should increase as the association between amount and timing strengthens, particularly when their effects are non-additive. Under some parameter combinations the conventional estimate may be attenuated; under others it may be inflated. Neither direction nor magnitude should be asserted in advance. The purpose of the simulation is to identify the regions of the parameter space in which each behaviour occurs and thereby determine whether departures from the classical attenuation baseline are large enough to matter in practice.

## A Time-Resolved, Context-Aware Construct

**Fig. 3:**
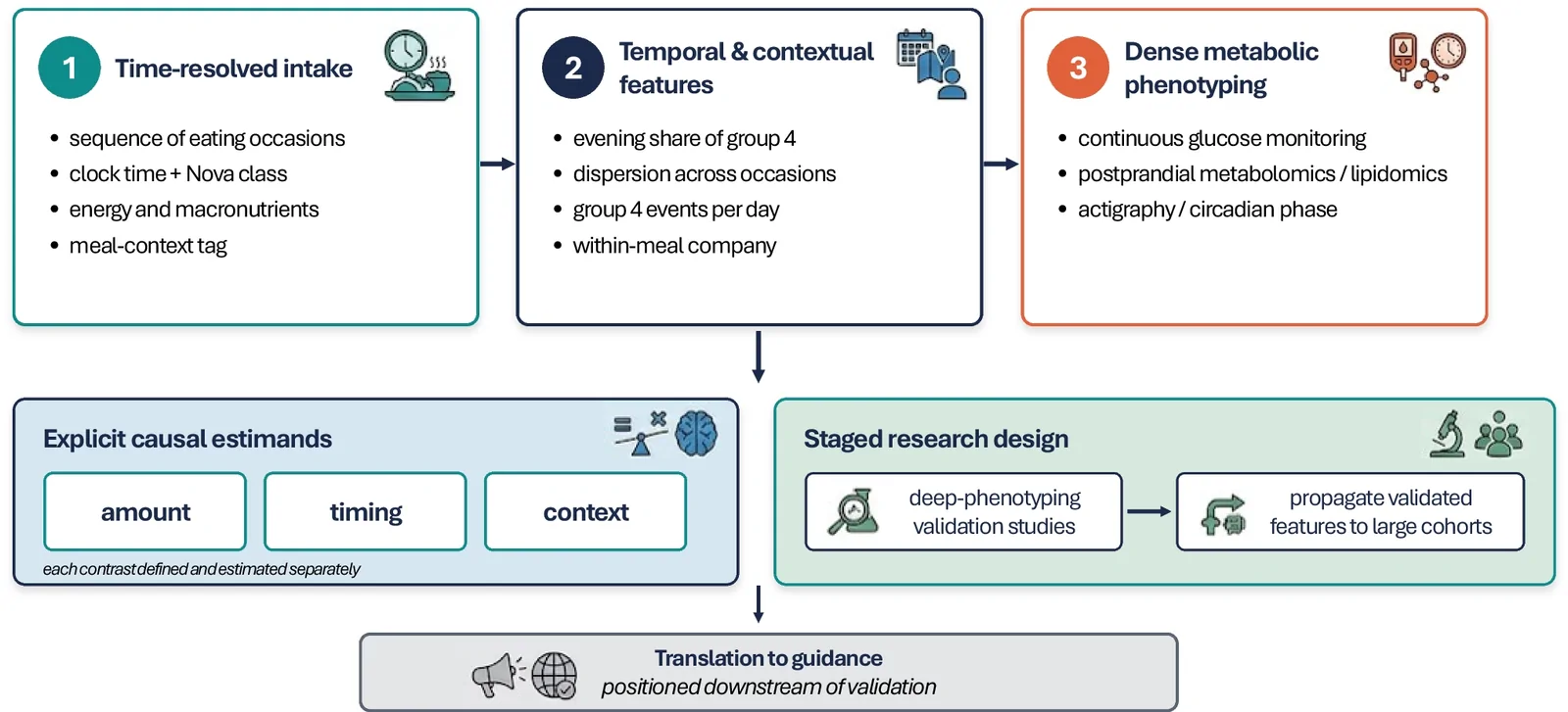
A time-resolved, context-aware exposure framework. Three layers, namely a sequence of tagged eating occasions, temporal and contextual summary features, and linkage to dense metabolic phenotyping by continuous glucose monitoring, postprandial metabolomics and actigraphy, feed explicit causal estimands defined separately for amount, timing and context. A staged design moves from deep-phenotyping validation to propagation of validated features into large cohorts, with translation to guidance positioned downstream of validation

The preceding argument identifies a potential deficiency in the dominant metric, not a justification for replacing it. Any alternative must therefore demonstrate incremental value rather than conceptual appeal. The appropriate response is therefore to construct the simplest representation that retains the discarded structure and then test whether it adds information beyond the scalar Fig. 3.

*A sequence before a summary.* The primary representation is an ordered sequence of eating occasions, not a replacement scalar. Each occasion records time, Nova classification, energy and macronutrient content, together with contextual information describing occasion type and the foods accompanying group 4 items. Time-stamped recalls already capture much of this information, while image-assisted and sensor-based methods can extend or validate it [70, 71].

Occasions must be defined consistently before features are derived. A pragmatic starting definition is any ingestion event supplying at least 50 kcal, with events separated by less than 15 min merged and occasion type assigned by participant report rather than clock time. Energy-containing beverages count as eating occasions. These thresholds are conventions, so analyses should report the sensitivity of derived features to plausible alternatives.

One exception is required. A 50 kcal threshold excludes artificially sweetened beverages and other non-caloric group 4 items. These products contribute negligible energy but may constitute a substantial component of group 4 consumption and still involve oral, cephalic-phase and gut-directed responses. The energy threshold should therefore define general eating occasions, whereas any consumption of a group 4 item should define a group 4 event, irrespective of energy content. Eating-occasion counts and group 4 event counts are consequently distinct and should be reported separately. Non-caloric items contribute to event-based features but, by definition, not to energy-weighted features. This asymmetry should be explicit.

*Features preserving structure.* From the sequence, summary features retain the relevant dimensions while remaining tractable: the circadian timing of group 4 energy, such as evening share or a clock-time centroid; dispersion across occasions; number of group 4 events per day; and the typical within-meal company of group 4 items. These are descriptive features rather than a formal model, and are computable from instruments already in use, so the construct can be applied retrospectively to existing cohorts.

A worked example illustrates the derived features. Suppose five occasions contribute 120, 200, 60, 150 and 315 kcal of group 4 energy at 07:30, 10:15, 13:00, 16:30 and 21:45, respectively. Total group 4 energy is 845 kcal. Of this, 315 kcal occurs after 18:00, giving an evening share of 0.37. The corresponding energy-weighted clock-time centroid is approximately 14:50 and the dispersion approximately 5.2 h. All five occasions are group 4 events; four are substantial group 4 occasions contributing at least 50 kcal of group 4 energy. These quantities can all be derived from a time-stamped recall, although alternative event definitions should be examined in sensitivity analyses.

*Validation programme.* The construct has value only if retained temporal or contextual features improve explanation or prediction beyond the daily group 4 fraction. Its incremental validity is the pivotal claim of this review and remains untested. Validation should proceed in three stages.

Stage one should establish biological sensitivity under controlled conditions. A randomised, isocaloric crossover feeding study could hold daily group 4 quantity and macronutrient content constant while varying the biological timing of an otherwise identical group 4 load. Continuous glucose monitoring and postprandial profiling would test whether the temporal contrast produces distinguishable responses. Appetite and subsequent energy intake could be examined as secondary outcomes. Sample-size requirements should be derived from the prespecified primary outcome and expected within-person variance rather than fixed in advance here.

Stage two should test incremental value in existing cohorts. Among cohorts retaining item-level time stamps, analyses should determine whether temporal features improve prediction of adiposity change beyond the conventional scalar and established covariates. Evaluation should emphasise out-of-sample performance, calibration, explained variance and information criteria rather than significance testing alone. Feature definitions and model comparisons should be preregistered to reduce outcome-guided selection.

Stage three should estimate explicit causal contrasts. Only features showing acceptable reliability and incremental value should proceed to causal analysis. Amount, timing and context interventions should then be defined separately, with their target populations, follow-up periods and identifying assumptions specified in advance through target-trial emulation [20, 74].

This sequence prevents the causal analysis from outrunning measurement validation: the construct must first show that it responds to controlled temporal variation, then that it adds information in observational data, before its features are interpreted as intervention targets.

### Measurement Error in Temporal and Contextual Features

Any gain from retaining temporal structure must be weighed against the error with which that structure is measured. The conventional scalar discards timing entirely, whereas the proposed construct recovers it from self-reported instruments whose limitations are well recognised. If a 24-h recall misstates what was consumed, it may also misstate when and under what circumstances it was consumed. The relevant comparison is therefore not between an error-prone construct and an error-free scalar, but between partial information measured with error and no representation of that information at all.

Three forms of error affect reported times. First, recall error displaces an eating occasion from its true temporal position, with misclassification plausibly increasing for unstructured eating and for occasions occurring further from the time of recall. Second, digit preference concentrates reported times on the hour and half-hour, producing a discretised representation of an underlying continuous distribution. Third, occasion mislabelling assigns an incorrect contextual category, for example when a substantial late intake is recorded as either a snack or a dinner according to individual convention rather than a consistent definition.

These errors affect the proposed features differently, and those differences have implications for study design. The clock-time centroid is an energy-weighted mean; independent displacement errors therefore tend to cancel, and approximately symmetric digit preference introduces little systematic bias. Among the proposed features, the centroid is likely to be the most robust. Dispersion, by contrast, is a second-moment measure and therefore inherits error variance directly. Reported dispersion will tend to exceed true dispersion, making comparisons between populations with different levels of reporting error difficult to interpret without correction. Evening share is likely to be the most vulnerable feature because it depends on threshold classification. An occasion occurring at 17:45 but reported at 18:00 crosses the boundary and changes the measure discontinuously. Error is therefore concentrated near the cut-point rather than distributed smoothly across the metric.

Four implications follow. First, features should be calculated at a temporal resolution the instrument can realistically support, favouring broad time bands, such as four six-hour windows, over minute-level precision. Second, threshold-based measures should be accompanied by sensitivity analyses across plausible boundary placements so that findings dependent on a particular cut-point can be identified. Third, repeat-administration substudies should be used to estimate the reliability of each feature directly, providing the error variance required both for correction and for the bias analyses described in Sect. "Specification of a Quantitative Bias Analysis". Fourth, where objective measures are available, including wearable ingestion sensors, image-assisted methods, or timestamped continuous glucose monitoring records, a validation subsample should be used to quantify disagreement with self-report.

The proposed construct therefore exchanges complete omission for imperfect measurement. Whether that exchange is beneficial depends on the reliability of the derived features, the strength of their association with the outcome and the extent to which they improve inference beyond daily amount. Moderate measurement error does not by itself invalidate a feature, but neither does conceptual relevance establish its utility. The validation programme must show that the recovered signal exceeds the error introduced in recovering it. If it does not, the case for adding that feature fails.

## Measurement and Validation Technologies

The proposed framework does not depend on a single technology. Rather, different tools address distinct layers of the measurement problem. Time-stamped recalls and food records provide the occasion sequence from which temporal and contextual features can be derived. Image-assisted methods and wearable ingestion sensors can improve or validate the timing and composition of those occasions [70, 71]. Actigraphy and sleep-timing measures permit clock time to be re-referenced to behavioural or circadian phase. Continuous glucose monitoring resolves occasion-level glycaemic responses [17, 72], while postprandial metabolomic and lipidomic profiling extends validation beyond glucose [18, 73]. Dietary biomarkers provide an objective complement for selected components of intake [74].

These technologies should therefore be deployed according to the stage of validation. Dense phenotyping is most useful in smaller mechanistic studies designed to establish biological sensitivity and measurement validity. Time-stamped self-report remains the more scalable instrument for testing incremental value in cohorts. The aim is not to require maximal measurement in every study, but to identify which features justify propagation into larger and less intensively characterised populations.

## Feasibility, Generalisability and Equity

The framework’s immediate feasibility depends less on new data collection than on whether existing occasion-level records have been retained. Multiple-pass 24-h recalls commonly record the time and name of each eating occasion, allowing temporal features to be reconstructed without further participant burden. In many analytic datasets, however, these fields are discarded when item-level records are reduced to nutrient totals. Recoverability must therefore be established cohort by cohort, often through access to source files rather than public releases. An audit of major cohorts retaining usable time fields would be an inexpensive first step.

Food-frequency questionnaires cannot recover occasion-level timing or context, which limits the framework’s retrospective reach. Because these instruments underpin a substantial proportion of the UPF evidence base, time-resolved measurement should not initially be treated as a condition for study validity or as a wholesale replacement for the scalar. The appropriate transitional strategy is to report both measures where possible, preserving comparability with existing evidence while the incremental value of temporal features is established.

Feasibility determines where the construct can be applied; equity determines how its results should be interpreted. Meal timing and occasion structure are shaped by shift work, caregiving, employment conditions and food access [68]. Temporal features may therefore capture not only individual behaviour but also the social conditions that produce it. This is both a strength and a source of confounding. The construct can make structurally patterned exposure visible, but observational timing estimates may partly reflect the circumstances associated with both eating patterns and adiposity.

Study design should address this problem directly. Within-person experiments hold stable social characteristics constant and are therefore the appropriate first test of timing effects. Analyses within occupationally or schedule-homogeneous groups can reduce between-person differences, while sensitivity analyses can assess the strength of residual confounding required to explain observed associations. These strategies cannot remove all uncertainty, and observational estimates should be interpreted accordingly.

The same reasoning shapes translation. If late or fragmented eating is produced by rostering, caregiving obligations or restricted food availability, the appropriate response may be structural rather than individual. Timing-aware evidence could support changes to shift rotation, workplace food provision and access to suitable foods outside conventional hours. Used in this way, the construct identifies where environmental intervention is warranted rather than assigning responsibility to individuals for schedules they cannot control.

## Implications for Obesity Practice

The proposed framework is not yet a clinical intervention, but it has immediate implications for dietary assessment.

Obesity practice routinely asks what patients eat and, often, how much. It less consistently records when UPFs are consumed, whether they occur within structured meals or isolated snacks, and what foods accompany them. Two patients with the same reported group 4 fraction may therefore differ in whether that intake is concentrated late, dispersed through repeated grazing, or embedded within composite meals. The conventional scalar cannot represent those distinctions.

A modest refinement is possible without assuming that timing-based intervention is effective Table 3. Clinicians can ask when the largest UPF contribution occurs, how many distinct eating occasions contain UPFs and whether those items are consumed alone or within meals. These questions may identify patterns that are feasible to modify and, equally importantly, patterns constrained by work, caregiving or access. Where continuous glucose monitoring is already clinically indicated, occasion-level trajectories may help relate reported patterns to individual glycaemic responses [17, 72].

**Table 3:** Time-resolved elements that can be added to a routine dietary history in obesity practice

What to ask	What it captures	Why it may matter	Possible response if modifiable
At what times does most ultra-processed intake occur?	Circadian placement of exposure	Later intake meets lower insulin sensitivity and predicts poorer weight-loss outcomes at matched intake	Shift a portion of intake earlier where the daily schedule permits
How many separate eating occasions occur in a day?	Occasion architecture	More occasions mean more postprandial events and weaker compensation	Consolidate grazing into fewer structured meals
Is intake concentrated after the evening meal or overnight?	Late and night eating	Night eating coincides with the least favourable circadian phase, and is common in shift work	Recompose or redistribute late occasions rather than target total intake alone
What accompanies ultra-processed items within a meal?	Co-ingestion and sequence	Fibre and protein eaten first blunt the subsequent glycaemic excursion	Reorder the meal, or add a vegetable or protein component first
Does the pattern reflect work, caring or access constraints?	Modifiability	Distinguishes patterns a patient can change from those set by circumstance	Direct effort to modifiable elements; avoid assigning responsibility for the rest

These refine assessment; none is a validated intervention (Sect. "Implications for Obesity Practice").

Three boundaries should remain explicit. First, no trial has established that timing-aware advice improves weight outcomes specifically among people with high UPF intake. These questions therefore refine assessment rather than define a validated intervention. Second, timing is often structurally constrained, so advice should distinguish modifiable patterns from those imposed by work, caregiving or food access. Third, redistribution is not a substitute for established obesity management. Total energy intake, overall dietary quality, physical activity, sleep, pharmacotherapy and metabolic surgery remain the primary treatment levers. The narrower proposition is that, if temporal features prove informative, the timing and context of the UPF intake that remains may offer an additional and more precisely defined target.

## Conclusion

UPF research has converged on the daily group 4 fraction as its dominant exposure metric. That measure is useful, but it is not the exposure itself. It is a summary that preserves amount while discarding when group 4 foods are consumed, how they are distributed across occasions and what accompanies them.

Evidence from circadian and postprandial research shows that timing and context can modify the metabolic response to food in general. It does not show that the same differentials apply to Nova group 4 foods, nor that retaining these dimensions will materially improve UPF-obesity research. The purpose of this review is not to assume that they do, but to specify how that proposition should be tested.

The required test is feasible. Existing time-stamped records can be used to reconstruct occasion-level sequences, quantitative bias analysis can determine whether temporal collapse produces more than classical attenuation, and controlled feeding with dense metabolic phenotyping can establish whether an identical group 4 load produces different responses across biological times and contexts. Only features that demonstrate acceptable reliability and incremental value should proceed to causal or clinical interpretation.

The immediate task is therefore not to replace the daily group 4 fraction, but to determine whether the information it discards matters. The field already possesses many of the necessary data, technologies and analytical methods. What remains is an empirical test of a question that has so far been assumed rather than examined: whether the dominant metric in UPF research has been averaging away information relevant to explanation, prediction and intervention.

## Key References

- Lane MM, Davis JA, Beattie S, Gómez-Donoso C, Loughman A, O'Neil A, Jacka F, Berk M, Page R, Marx W, Rocks T (2021) Ultraprocessed food and chronic noncommunicable diseases: A systematic review and meta-analysis of 43 observational studies. Obes Rev 22 (3):e13146. https://doi.org/10.1111/obr.13146.
  - ○ The most comprehensive synthesis of UPF-health associations to date, and the empirical foundation this review accepts before turning to how the exposure is measured.

- Hall KD, Ayuketah A, Brychta R, Cai H, Cassimatis T, Chen KY, Chung ST, Costa E, Courville A, Darcey V, Fletcher LA, Forde CG, Gharib AM, Guo J, Howard R, Joseph PV, McGehee S, Ouwerkerk R, Raisinger K, Rozga I, Stagliano M, Walter M, Walter PJ, Yang S, Zhou M (2019) Ultra-Processed Diets Cause Excess Calorie Intake and Weight Gain: An Inpatient Randomized Controlled Trial of Ad Libitum Food Intake. Cell Metab 30 (1):67–77 e63. https://doi.org/10.1016/j.cmet.2019.05.008.
  - ○ The pivotal controlled feeding trial showing ultra-processed diets promote overconsumption beyond average nutrient composition; the anchor for both mechanistic enquiry and the present measurement argument.

- Baker P, Slater S, White M, Wood B, Contreras A, Corvalán C, Gupta A, Hofman K, Kruger P, Laar A, Lawrence M, Mafuyeka M, Mialon M, Monteiro CA, Nanema S, Phulkerd S, Popkin BM, Serodio P, Shats K, Van Tulleken C, Nestle M, Barquera S (2025) Towards unified global action on ultra-processed foods: understanding commercial determinants, countering corporate power, and mobilising a public health response. The Lancet 406 (10,520):2703–2726. 10.1016/S0140-6736(25)01567–3.
  - ○ Sets out the current global policy mobilisation on ultra-processed foods, the context in which exposure definition now carries regulatory weight.

- Berry SE, Valdes AM, Drew DA, Asnicar F, Mazidi M, Wolf J, Capdevila J, Hadjigeorgiou G, Davies R, Al Khatib H, Bonnett C, Ganesh S, Bakker E, Hart D, Mangino M, Merino J, Linenberg I, Wyatt P, Ordovas JM, Gardner CD, Delahanty LM, Chan AT, Segata N, Franks PW, Spector TD (2020) Human postprandial responses to food and potential for precision nutrition. Nat Med 26 (6):964–973. 10.1038/s41591-020–0934-0
  - ○ Large-scale postprandial phenotyping revealing meal-specific and person-specific metabolic signatures; the outcome-side technology the proposed construct relies upon.

- Thiébaut ACM, Boshuizen HC, Midthune D, Wallace MP, Kipnis V, Gustafson P, of the international STRengthening Analytical Thinking for Observational Studies initiative oboME, Group MT (2026) Five misconceptions about categorizing exposure variables measured with error in epidemiological research. Am J Epidemiol. 10.1093/aje/kwag071.
  - ○ Recent corrective on how error-prone exposure variables behave under categorisation and aggregation, directly relevant to the collapse examined here.

- Louie JCY (2026) The gut–brain–adipose axis in ultra-processed food and obesity: a mechanistic synthesis and its implications for food classification. Current Obesity Reports Accepted manuscript.
  - ○ Companion review in this journal. Establishes the mechanistic case for attribute-aware refinement of Group 4 on compositional grounds; cited throughout to demarcate scope, since the present review holds composition fixed and addresses temporal aggregation instead.

- Touvier M, da Costa Louzada ML, Mozaffarian D, Baker P, Juul F, Srour B (2023) Ultra-processed foods and cardiometabolic health: public health policies to reduce consumption cannot wait. BMJ 383:e075294. 10.1136/bmj-2023–075294.
  - ○ Sets out the policy agenda now being built on the current exposure metric, which is what makes its adequacy urgent

- Braesco V, Souchon I, Sauvant P, Haurogne T, Maillot M, Feart C, Darmon N (2022) Ultra-processed foods: how functional is the Nova system? Eur J Clin Nutr 76 (9):1245–1253. 10.1038/s41430-022–01099-1.
  - ○ Documents classification uncertainty in Nova coding, against which any time-resolved construct must be benchmarked.

- Cordova R, Kliemann N, Huybrechts I, Rauber F, Vamos EP, Levy RB, Wagner KH, Viallon V, Casagrande C, Nicolas G, Dahm CC, Zhang J, Halkjær J, Tjønneland A, Boutron-Ruault MC, Mancini FR, Laouali N, Katzke V, Srour B, Jannasch F, Schulze MB, Masala G, Grioni S, Panico S, van der Schouw YT, Derksen JWG, Rylander C, Skeie G, Jakszyn P, Rodriguez-Barranco M, Huerta JM, Barricarte A, Brunkwall L, Ramne S, Bodén S, Perez-Cornago A, Heath AK, Vineis P, Weiderpass E, Monteiro CA, Gunter MJ, Millett C, Freisling H (2021) Consumption of ultra-processed foods associated with weight gain and obesity in adults: A multi-national cohort study. Clin Nutr 40 (9):5079–5088. 10.1016/j.clnu.2021.08.009.
  - ○ Multinational cohort evidence extending UPF associations to cardiometabolic multimorbidity.

## Data Availability

No datasets were generated or analysed during the current study.
